# Parent-of-origin effects in the life-course evolution of cardiometabolic traits

**DOI:** 10.1007/s00125-025-06396-5

**Published:** 2025-04-02

**Authors:** Rucha Wagh, Gad Hatem, Jonas Andersson, Pooja Kunte, Souvik Bandyopadhyay, Chittaranjan S. Yajnik, Rashmi B. Prasad

**Affiliations:** 1 Diabetes Unit, Kamalnayan Bajaj Diabetology Research Centre, King Edward Memorial Hospital and Research Centre, Pune, India; 2 Symbiosis School of Biological Sciences, Symbiosis International (Deemed University), Pune, India; 3 Department of Clinical Sciences, Diabetes and Endocrinology, Lund University, Malmö, Sweden; 4 Cytel Inc., Cambridge, MA USA; 5 Institute of Molecular Medicine Finland, Helsinki University, Helsinki, Finland

**Keywords:** Genetics, Life-course programming, Macronutrients, Parent-of-origin, Sex-specific parental effects, Type 2 diabetes

## Abstract

**Aims/hypothesis:**

Cardiometabolic traits are heritable, and some display parent-of-origin effects, which indicates preferential inheritance from one parent or parental bias. Most studies of these phenomena have focused on adult populations. We aimed to investigate the heritability and parent-of-origin effects on cardiometabolic traits in a birth cohort with serial measurements to determine whether these patterns emerged early in life.

**Methods:**

The Pune Maternal Nutrition Study comprises a birth cohort in which offspring and parents were studied from birth and followed up for 24 years. We investigated parent-of-origin effects on cardiometabolic traits cross-sectionally at available timepoints using linear regression, and longitudinally across the life course using mixed-effect regression. Maternal and paternal effects on offspring phenotype were modelled after adjusting for age, sex and BMI. Parent-of-origin effects were calculated based on the difference between maternal and paternal effects. We also investigated these effects in another birth cohort, that of the Pune Children’s Study. Genetic parent-of-origin effects were assessed using generalised estimating equations after taking the parental origin of the alleles into account.

**Results:**

Birthweight showed a maternal parent-of-origin effect. At 24 years, maternal bias was seen for some obesity-related traits for daughters, while paternal bias was seen for WHR in sons. A shift from paternal bias at 6 years to maternal bias at 24 years for the skinfold thickness was observed in daughters. Fasting glucose and lipids showed maternal bias at 6, 12 and 24 years. For fasting insulin and HOMA2-S, a negative maternal effect at 6 years transitioned to a positive one at 12 years. For HOMA2-B, a paternal effect at 6 years transitioned to a maternal one at 12 years, and this remained so at 24 years. Some of these findings were also observed in the cohort from the Pune Children’s Study. Longitudinal modelling revealed stronger paternal effects over time for fasting insulin and HOMA indices but maternal effects for glucose and lipids, reflecting their cumulative effect over time. Genetic variants at the *KCNQ1* locus showed a maternal parent-of-origin effect on birthweight, on HOMA2-B at 12 years, and on lipids at 6 and 12 years.

**Conclusions/interpretation:**

Our study provides proof of concept of the existence of parent-of-origin effects on cardiometabolic traits from birth, through childhood and puberty, until adult age. Our results indicate a predominantly maternal influence on intrauterine, pubertal and reproductive-age metabolism in the offspring. While the longitudinal analysis indicated a maternal bias for the macronutrients (glucose and lipids), and a paternal bias for glucose–insulin metabolism, the cross-sectional analysis revealed a transition between parental influence across physiological stages. This dynamic relationship may have its origins in the life-history theory of evolution, and could inform strategies for primordial prevention aimed at curbing the rising burden of cardiometabolic disease. Further studies are needed to determine the mechanisms underlying such effects.

**Graphical Abstract:**

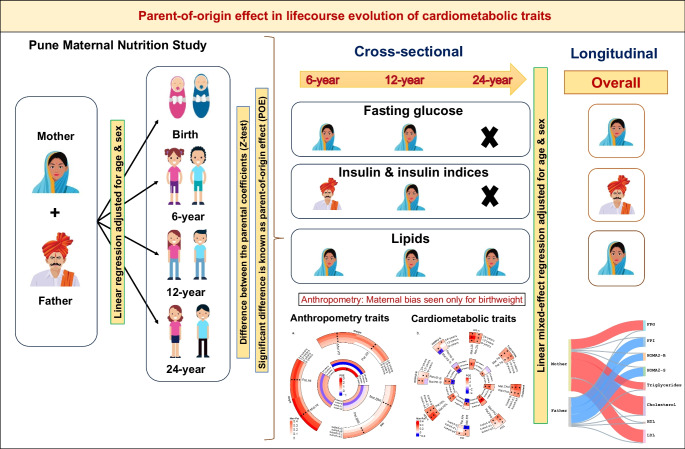

**Supplementary Information:**

The online version contains peer-reviewed but unedited supplementary material available at 10.1007/s00125-025-06396-5.


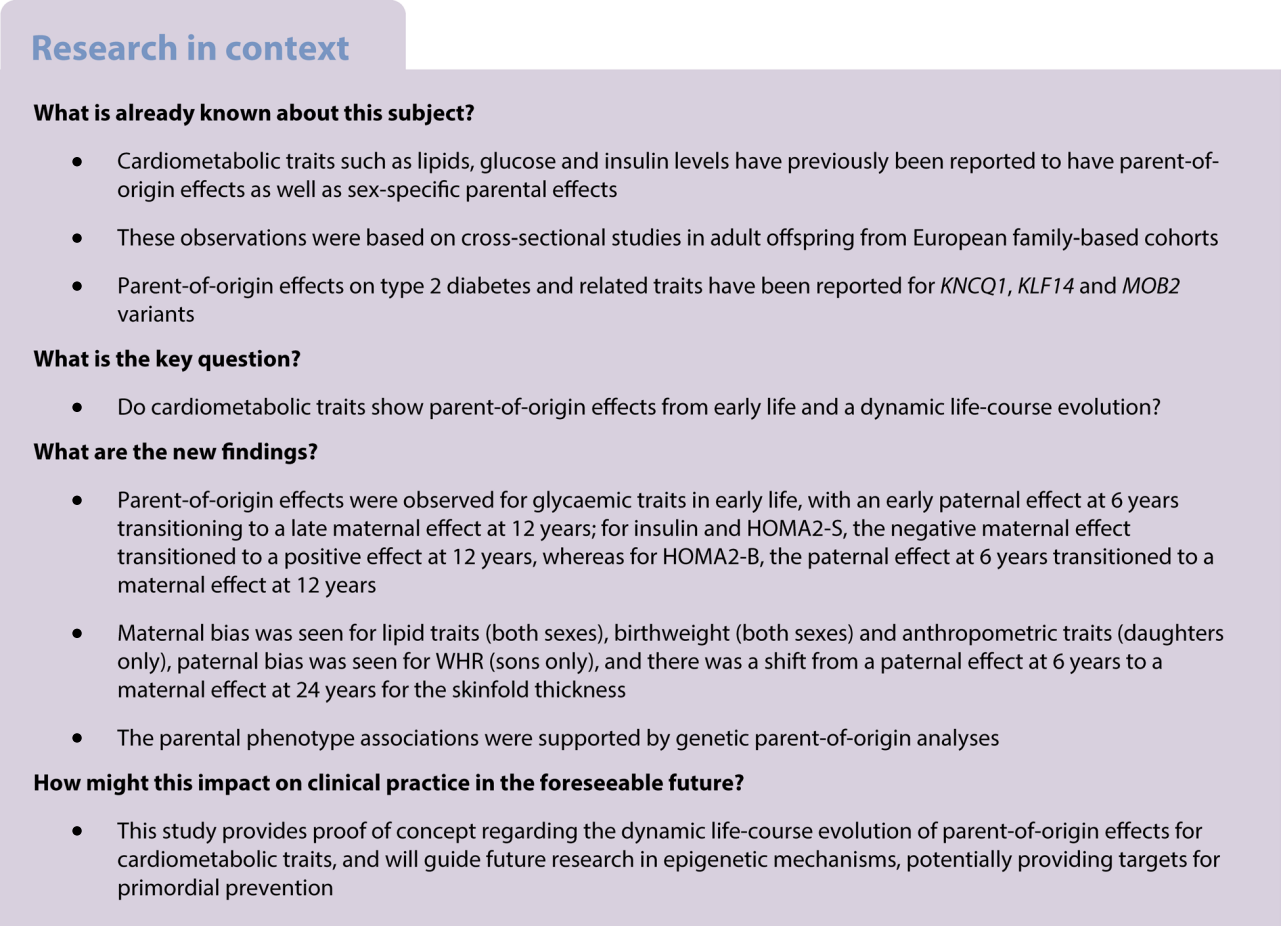


## Introduction

Human traits and diseases are the consequence of a complex interplay between genetics and environment. Heritability measures how much of the variation in a trait within a population is due to genetic factors. Anthropometric and metabolic traits have been shown to be heritable to varying degrees [1, 2]. Genetic association studies have identified a number of variants that are associated with these traits; however, the proportion of heritability attributed to these variants is rather limited [3, 4].

In a classic Mendelian pattern of transmission, a trait may be inherited from both the parents equally; it is also possible that it may be inherited preferentially from one of the parents, while the contribution of the other parent may be low, neutral or even opposite. Such effects (whereby the expression of the phenotype in the offspring depends upon which parent they are inherited from) are termed parent-of-origin effects. These effects may be attributed to genetic imprinting, intrauterine effects or maternally inherited mitochondrial genes [5]. The significance of such effects in the aetiology of type 2 diabetes and obesity has been emphasised previously [2, 6–8]. Type 2 diabetes shows a preferential maternal transmission [2, 7], and a substantial component may originate in the intrauterine period. Several studies have demonstrated that early-life exposures can influence developmental programming and increase the risk of cardiometabolic disorders in later life [9–11].

Parent-of-origin as well as offspring sex-specific parental effects have been reported for anthropometric measurements, insulin secretion and cholesterol levels [1, 2, 12], and these results are supported by genetic associations [8, 13, 14]. For instance, the sons of mothers with type 2 diabetes had lower insulin concentrations compared with the sons of fathers with the same condition, while the daughters of mothers with type 2 diabetes had lower HDL-cholesterol levels compared with the daughters of fathers with type 2 diabetes [2].

The previous studies showing parent-specific effects on cardiometabolic outcomes were performed in adult offspring of European origin and comprised a cross-sectional design. In the present study, we aimed to investigate the role of early-life programming and life-course tracking on cardiometabolic traits from early life to young adulthood, to examine whether these parental effects manifested at an early age. Our objective was to gain insights into intergenerational transmission of metabolic traits and chronic disease risk to identify possible earlier points of intervention, with the overarching aim of primordial prevention.

Primordial prevention may target the preconception period, pregnancy or early childhood to reduce risk factors for non-communicable diseases. The preferred period is preconception, and classic success stories include the use of folic acid (with or without other micronutrients) in prevention of neural tube defects, and strict control of maternal hyperglycaemia to prevent congenital anomalies in pregnancies affected by diabetes [15, 16]. Such interventions are effective largely due to epigenetic modifications during intergenerational transmission [15, 16]. Recently, there has been growing acknowledgement of the role of epigenetic mechanisms occurring in sperm cells [17–19]. Epigenetic programming and reprogramming continue in postnatal life, although the time windows become progressively narrower. Modifying the family environment to reduce obesity and related disorders is an example of postnatal intervention. Overall, preconception measures primarily target mothers (and increasingly fathers), while postnatal efforts focus on families.

The Pune Maternal Nutrition Study (PMNS), which studies a well-characterised prospective birth cohort from India, provides a unique opportunity to investigate such effects in parent–offspring trios in a life-course model. In this study, we investigated the heritability and parent-of-origin effects for anthropometric, glycaemic, insulin-related and lipid traits in the PMNS birth cohort, with follow-up from birth through puberty till adulthood. Parent–offspring associations and transitions of parent-specific effects on offspring measures across childhood were assessed cross-sectionally (at each available timepoint) as well as longitudinally (across time). We also assessed these effects in another birth cohort, that of the Pune Children’s Study (PCS), with similar follow-up. Genetic variants that were previously shown to have parent-of-origin effects on cardiometabolic traits were assessed for similar effects at each available timepoint in the PMNS to better understand genetic contributions vs non-genetic contributions (e.g. the intrauterine environment).

## Methods

### Cohort characteristics for the PMNS

**Fig. 1:**
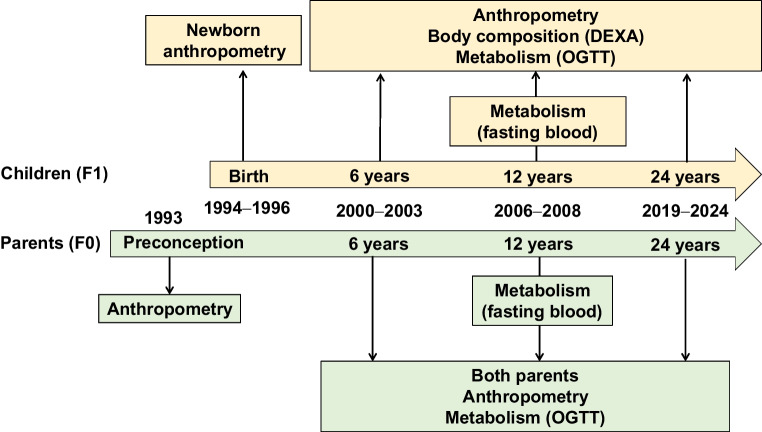
Study design for the PMNS. DEXA, dual-energy x-ray absorptiometry

The PMNS (Fig. 1 and electronic supplementary material [ESM] Fig. 1) was a community-based preconceptional rural birth cohort study established in 1993 in six villages near Pune, India, to prospectively study associations of maternal nutritional status with fetal growth and later diabetes risk in the offspring. Married, non-pregnant women aged 15–40 years (*N*=2466) were invited to participate, and those who consented and became pregnant between 1994 and 1996 (singleton pregnancy <21 weeks of gestation) were recruited into the study. They were followed up, together with their spouses (F0 generation), and subsequently their offspring (F1 generation). The majority of the families belonged to the Hindu religion. The prevalence of gestational diabetes was <1% based on WHO 1985 criteria (the criteria that were applicable at the time of the visit [20]). Growth and metabolism-related measurements were performed on the parents and the offspring at birth and every 6 years thereafter (ESM Table 1). Final analysis for heritability and parent-specific effects in this study was performed on the participants with complete data at each timepoint. The cohort was representative of the population from which it was derived.

### Anthropometric and clinical measurements in parents and offspring

Body size (anthropometric traits), glucose tolerance (as assessed using a 75 g OGTT) and corresponding insulin concentrations were measured in both parents at the time of the initial visit and at the 6- and 24-year follow-up visits. Only body size measurements and a fasting blood test were available at the 12-year follow-up visit.

Newborn anthropometric measurements comprising weight, length, abdominal circumference and skinfold thickness were obtained within 72 h birth. Comprehensive assessments of body composition and glucose and insulin concentrations in the offspring were performed at 6, 12 and 24 years. Total fat and lean mass and body fat percentage were measured using dual-energy x-ray absorptiometry (Lunar DPX-IQ 240 pencil beam machine, Lunar Corporation). BMI was calculated as weight (kg)/square of height (m^2^), WHR was calculated as waist circumference (cm)/hip circumference (cm), and the body roundness index was calculated as described previously [21].

For the biochemistry measurements, participants (both parents and offspring) arrived at the Diabetes Unit of the King Edward Memorial Hospital, Pune, India, the evening before, had a standardised dinner, and fasted overnight. In the morning, a fasting blood sample was collected. At 6 years, an OGTT was performed, using 1.75 g/kg anhydrous glucose. At 12 years, only a fasting sample was collected. At 24 years, a full OGTT (using 75 g anhydrous glucose) was repeated.

Glucose was measured using the glucose oxidase/peroxidase method, and specific insulin by ELISA (ESM Table 1). HOMA2-S and HOMA2-B were calculated using data from the fasting samples and the calculation provided on the iHOMA2 website (https://www.phc.ox.ac.uk/research/technology-outputs/ihoma2) [22]. Lipid measurements (triglycerides, total cholesterol and HDL-cholesterol) were made using standardised enzymatic assays, and LDL-cholesterol was calculated using Friedewald’s formula.

### PCS birth cohort

The PCS was a hospital-based urban birth cohort of approximately 400 children born in the King Edward Memorial Hospital, Pune, India, between 1987 and 1989, and their parents. All children born in the above time frame based on hospital registers were invited to participate. The majority of the families belonged to the Hindu religion. Serial measurements for anthropometric traits were obtained at 4, 8 and 21 years, and for cardiometabolic traits at 8 and 21 years. The methods of measurements were the same as described above for the PMNS. The cohort was representative of the population from which it was derived.

### Statistical analysis

Heritability and parent-of-origin effects for cardiometabolic traits were assessed between the F0 and F1 generations across the various timepoints in the PMNS cohort (6, 12 and 24 years), and also for anthropometric traits at birth. Skewed variables were transformed using logarithmic or inverse normal transformations. Heritability was estimated using regression models, with offspring phenotype as the outcome and mid-parental phenotype (mean) as the predictor, adjusted for age and sex of both parents and offspring, and presented as β coefficients (with SE) and corresponding *p* values. Maternal- and paternal-specific effects were investigated cross-sectionally (at each timepoint) using regression models to assess evolution of the effect over time, and longitudinally using mixed-effect regression models to assess the overall effect on the offspring, using intra-family and inter-observation correlations as random effects. All models were adjusted for age and sex for anthropometric traits and for age, sex and BMI of both parents and offspring for cardiometabolic traits. All reported associations from the regression models were significant after multiple testing using the Bonferroni method (adjusted *p* value threshold ≤0.001). Parent-of-origin effects were tested by calculating the difference in maternal and paternal regression coefficients using the Wald test: $Z=(b1-b2)/\surd [\text{SE}b{1}^{2}+\text{SE}b{2}^{2}-cov\left(b1\times b2\right)$], where *b*1 and *b*2 are regression coefficients, SE are the corresponding standard errors and *cov* refers to corresponding covariances, and are expressed as *Z* values with the corresponding *p* value using the cumulative probability function $p=2{\times}[{1-pnorm(abs(|Z|))}]$. Values of *p*<0.05 were considered statistically significant.

### Genetic parent-of-origin analysis

#### GWAS quality control and imputation

Genome-wide genotyping data were generated for the PMNS trios (mother, father and offspring) using Affymetrix SNP 6.0 chips. SNPs were excluded if the missingness was >5%, the minor allele frequency was <1% and the Hardy–Weinberg equilibrium *p* value was <0.05. Further quality control included allele checks, call rate and Mendelian errors. Imputation was performed on the TOPMED imputation server using the TOPMED r3 (GRCh38/hg38) as a reference panel with Eagle version 2.4 phasing and Minimac4 imputation [23–25]. SNPs of interest were extracted based on having imputation scores >0.4.

#### Parent-of-origin analysis

The rs2237892 SNP at the *KCNQ1* locus, the rs4731702 SNP at the *KLF14* locus and the rs2334499 SNP at the *MOB2* locus have previously been shown to display parent-of-origin effects on type 2 diabetes risk [8, 14, 26]. The *KCNQ1* variants have also been shown to be associated with insulin secretion, fasting glucose, birthweight and placental weight, whereas variants at *KLF14* show associations with lipid levels and variants at *MOB2* show associations with insulin sensitivity [26–30]. These SNPs were assessed for their association with the respective traits (where available) in a parent-of-origin manner for each allele at available timepoints. First, the parental origin was determined using a custom R script taking into account the parental genotypes. Then the generalised estimating equations R package geepack [31] was used, with independence as the correlation structure for major allele A and minor allele B, where m is the maternally inherited allele and p is the paternally inherited allele, for the following models: maternal, ApBm vs AA; paternal, AmBp vs AA; parent-of-origin effects, ApBm vs AmBp. Offspring sex and age were used as covariates where applicable, and all traits of interests were first log-transformed and then converted to *z* scores. A *p* value <0.05 was considered to be statistically significant.

### Ethics statement

The study has been approved by the village leaders and the institutional committee (King Edward Memorial Hospital Research Centre Ethics Committee) at all timepoints for the PMNS, and by the institutional committee (King Edward Memorial Hospital Research Centre Ethics Committee) at all timepoints for the PCS. Participants ≥ 18 years of age signed an informed consent form. Children <18 years of age provided assent, together with parental consent.

## Results

### Parent-of-origin and offspring sex-specific parental effects on anthropometric and cardiometabolic traits

The PMNS birth cohort comprises approximately 700 parent–offspring trios with serial measurements for anthropometric traits at birth and at 6, 12 and 24 years old, and for cardiometabolic traits at 6, 12 and 24 years old (Fig. 1). The parents were on average short and underweight (based on WHO criteria for stunting and obesity [32–34]) before and during pregnancy. The offspring achieved greater height and weight than their parents, and had substantially higher levels of circulating macronutrients as adults (ESM Tables 2A and 2B). To investigate the proportion of offspring phenotypic traits attributable to parent phenotype variation through the life course, we calculated heritability estimates for cardiometabolic traits at available timepoints (ESM Tables 3 and 4). We next examined whether there was an association between the trait of the offspring and the trait of the mother (maternal effect) or between the trait of the offspring and the trait of the father (paternal effect). If offspring traits showed a significantly stronger association with the mother’s traits compared with the father’s, this would indicate a maternal bias or maternal parent-of-origin effect, and similarly for paternal bias (paternal parent-of-origin effect).

#### Anthropometric traits

**Fig. 2:**
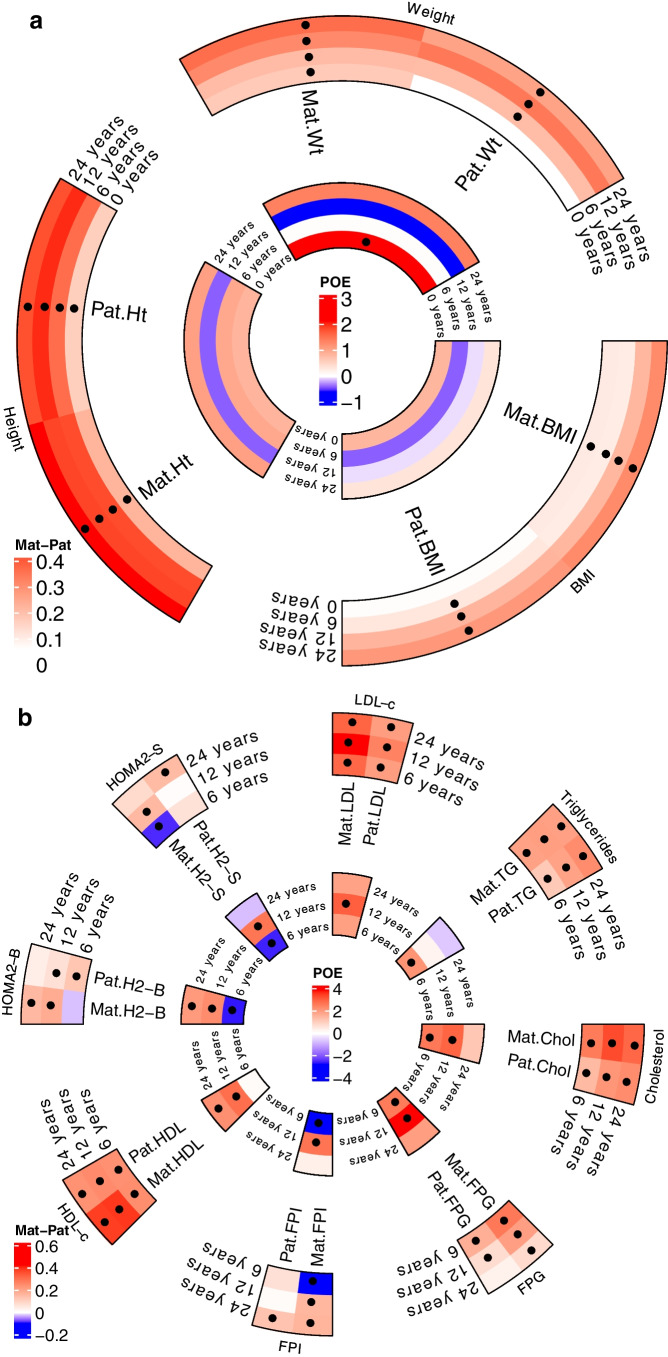
Circular heatmaps for (**a**) anthropometric traits and (**b**) metabolic traits, representing the phenotype associations between maternal traits or paternal traits and the offspring traits at the various timepoints. The outer circle represents the β coefficients for mother–offspring associations and father–offspring associations. The inner circle represents the parent-of-origin effects expressed as *Z* values. The black dots indicate a significant *p* value (*p*<0.05). Wt, weight; Ht, height; Chol, cholesterol; FPG, fasting plasma glucose; FPI, fasting plasma insulin; H2-S, HOMA2-S; H2-B, HOMA2-B; HDL-c and HDL, HDL-cholesterol; LDL-c and LCL, LDL-cholesterol; TG, triglycerides; Mat, maternal; Pat, paternal; POE, parent-of-origin effects

**Fig. 3:**
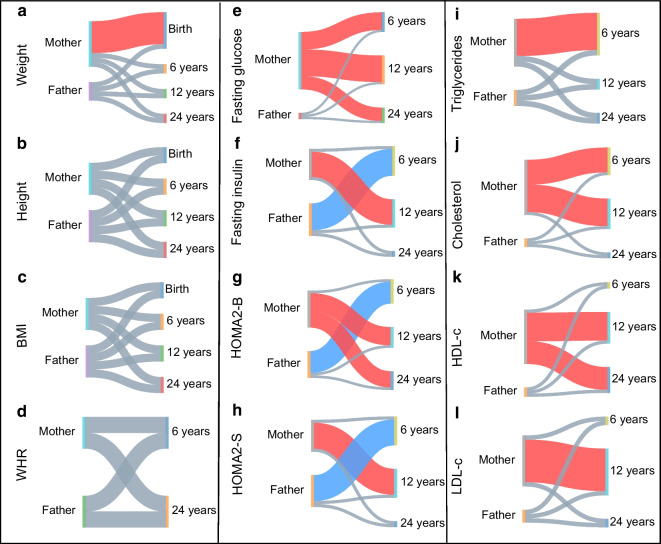
Individual Sankey diagrams representing the parent-of-origin effects (expressed as *Z* values) separately for each phenotype at each available timepoint: (**a**–**d**) anthropometric traits; (**e**–**h**) glycaemic traits; (**i**–**l**) lipids. A significant maternal bias is indicated in red, and a significant paternal bias is indicated in blue. Insignificant parent-of-origin effects are represented in grey. HDL-c, HDL-cholesterol; LDL-c, LDL-cholesterol

Most of the traits were heritable at all timepoints, with an increasing trend in the parental effect from birth to 24 years (Fig. 2a and ESM Tables 3 and 4). A significant maternal parent-of-origin effect was observed for birthweight when male and female offspring were analysed together (Table 1, Figs 2a and 3a). Daughters showed a significant paternal bias for the sum of skinfolds at 6 years, but a stronger maternal effect at 24 years. At 24 years, daughters also showed a maternal bias for weight, BMI, waist and hip circumference and WHR, but a paternal bias for WHR was observed for sons (Table 1 and ESM Table 5).

**Table 1:** Parent-of-origin test for offspring anthropometric traits

	Mother–child associations		Father–child associations		
	β (SE)	*p* value	β (SE)	*p* value	*Z* value (*p* value)
At birth					
All offspring	*N*=745		*N*=705		
Weight (kg)	0.176 (0.045)	0.000104191	0.058 (0.034)	0.094857416	2.10 (0.036)
Height (cm)	0.223 (0.053)	3.42 × 10^−05^	0.165 (0.048)	0.000645348	0.81 (0.418)
BMI (kg/m^2^)	0.106 (0.042)	0.012537531	0.063 (0.033)	0.054005535	0.80 (0.421)
Sons	*N*=388		*N*=368		
Weight (kg)	0.158 (0.058)	0.006646421	0.032 (0.043)	0.463051919	1.75 (0.081)
Height (cm)	0.227 (0.075)	0.00257224	0.155 (0.064)	0.01625536	0.74 (0.460)
BMI (kg/m^2^)	0.118 (0.057)	0.037943635	0.039 (0.044)	0.374421164	1.10 (0.272)
Daughters	*N*=357		*N*=337		
Weight (kg)	0.235 (0.072)	0.001322832	0.100 (0.056)	0.07317818	1.48 (0.139)
Height (cm)	0.230 (0.074)	0.00219911	0.203 (0.073)	0.005667585	0.26 (0.793)
BMI (kg/m^2^)	0.113 (0.064)	0.080784399	0.094 (0.048)	0.05364251	0.23 (0.818)
At 6 years					
All offspring	*N*=690		*N*=655		
Weight (kg)	0.207 (0.027)	1.81 × 10^−13^	0.206 (0.025)	2.36 × 10^−15^	0.02 (0.981)
Height (cm)	0.421 (0.042)	2.53 × 10^−22^	0.366 (0.041)	2.51 × 10^−18^	0.95 (0.345)
BMI (kg/m^2^)	0.101 (0.019)	7.06 × 10^−08^	0.112 (0.017)	8.81 × 10^−11^	−0.44 (0.658)
Sons	*N*=363		*N*=342		
Weight (kg)	0.193 (0.035)	9.11 × 10^−08^	0.206 (0.033)	1.18 × 10^−09^	−0.28 (0.782)
Height (cm)	0.458 (0.058)	5.89 × 10^−14^	0.338 (0.054)	1.61 × 10^−09^	1.50 (0.133)
BMI (kg/m^2^)	0.093 (0.024)	0.00012994	0.096 (0.022)	2.63 × 10^−05^	−0.10 (0.923)
Daughters	*N*=327		*N*=313		
Weight (kg)	0.224 (0.044)	5.54 × 10^−07^	0.208 (0.040)	2.88 × 10^−07^	0.26 (0.792)
Height (cm)	0.380 (0.060)	8.12 × 10^−10^	0.415 (0.062)	1.10 × 10^−10^	−0.41 (0.684)
BMI (kg/m^2^)	0.113 (0.029)	0.000122813	0.131 (0.026)	7.15 × 10^−07^	−0.47 (0.638)
At 12 years					
All offspring	*N*=658		*N*=597		
Weight (kg)	0.312 (0.039)	4.93 × 10^−15^	0.345 (0.037)	1.32 × 10^−19^	−0.63 (0.531)
Height (cm)	0.430 (0.049)	2.08 × 10^−17^	0.460 (0.047)	7.59 × 10^−21^	−0.44 (0.660)
BMI (kg/m^2^)	0.217 (0.031)	5.02 × 10^−12^	0.221 (0.030)	5.98 × 10^−13^	−0.09 (0.929)
Sons	*N*=342		*N*=314		
Weight (kg)	0.263 (0.047)	4.57 × 10^−08^	0.332 (0.044)	4.10 × 10^−13^	−1.08 (0.280)
Height (cm)	0.522 (0.067)	7.76 × 10^−14^	0.423 (0.063)	9.97 × 10^−11^	1.09 (0.276)
BMI (kg/m^2^)	0.152 (0.037)	5.03 × 10^−05^	0.208 (0.036)	1.53 × 10^−08^	−1.09 (0.278)
Daughters	*N*=316		*N*=283		
Weight (kg)	0.364 (0.063)	2.22 × 10^−08^	0.360 (0.061)	1.01 × 10^−08^	0.04 (0.966)
Height (cm)	0.326 (0.072)	9.56 × 10^−06^	0.486 (0.071)	6.09 × 10^−11^	−1.58 (0.115)
BMI (kg/m^2^)	0.284 (0.050)	2.79 × 10^−08^	0.238 (0.048)	1.49 × 10^−06^	0.66 (0.511)
At 24 years					
All offspring	*N*=462		*N*=357		
Weight (kg)	0.365 (0.056)	2.56 × 10^−10^	0.259 (0.057)	6.75 × 10^−06^	1.34 (0.182)
Height (cm)	0.482 (0.048)	1.02 × 10^−20^	0.415 (0.043)	1.10 × 10^−19^	1.03 (0.303)
BMI (kg/m^2^)	0.309 (0.058)	2.36 × 10^−07^	0.283 (0.063)	1.20 × 10^−05^	0.30 (0.761)
Sons	*N*=253		*N*=205		
Weight (kg)	0.328 (0.073)	1.23 × 10^−05^	0.353 (0.071)	1.51 × 10^−06^	−0.24 (0.808)
Height (cm)	0.505 (0.064)	2.05 × 10^−13^	0.446 (0.056)	1.39 × 10^−13^	0.69 (0.489)
BMI (kg/m^2^)	0.253 (0.072)	0.000642941	0.390 (0.075)	6.88 × 10^−07^	−0.57 (0.571)
Daughters	*N*=209		*N*=152		
Weight (kg)	0.401 (0.086)	6.77 × 10^−06^	0.094 (0.092)	0.31322184	2.44 (0.015)
Height (cm)	0.447 (0.074)	1.43 × 10^−08^	0.376 (0.068)	1.41 × 10^−07^	0.71 (0.480)
BMI (kg/m^2^)	0.381 (0.094)	9.00 × 10^−05^	0.094 (0.110)	0.392542665	1.98 (0.048)

Associations between offspring anthropometric measurements with that of each of the parents are presented as β values and SE. Differences between maternal and paternal effects are presented as *Z* values and *p* values. The *Z* value shows the difference between regression coefficients. All the regression models were adjusted for age and sex

#### Glucose and insulin indices

For fasting glucose concentrations, the maternal effects were significantly stronger than the paternal effects at 6, 12 and 24 years (Figs 2b and 3e, f and Table 2), in all offspring combined as well as sons only. These effects were seen only at 12 years old in daughters.

**Table 2:** Parent-of-origin effects for cardiometabolic traits

	6 years					12 years					24 years				
	MC		FC			MC		FC			MC		FC		
	β (SE)	*p* value	β (SE)	*p* value	*Z* value (*p* value)	β (SE)	*p* value	β (SE)	*p* value	*Z* value (*p* value)	β (SE)	*p* value	β (SE)	*p* value	*Z* value (*p* value)
All offspring	*N*=690		*N*=655			*N*=658		*N*=597			*N*=462		*N*=357		
Fasting glucose	0.287 (0.034)	1.99 × 10^−16^	0.174 (0.028)	7.58 × 10^−10^	2.59 (0.010)	0.212 (0.030)	4.89 × 10^−12^	0.078 (0.018)	1.71 × 10^−05^	3.84 (0.000)	0.082 (0.021)	1.19 × 10^−04^	0.030 (0.017)	0.066	1.92 (0.055)
Fasting insulin	−0.090 (0.041)	0.0275	0.069 (0.042)	0.1	−2.72 (0.007)	0.158 (0.041)	1.333 × 10^–04^	0.010 (0.037)	0.784	2.68 (0.007)	0.165 (0.075)	0.029	0.132 (0.060)	0.034	0.31 (0.758)
HOMA2-B	−0.023 (0.040)	0.57	0.128 (0.040)	0.001	−2.66 (0.008)	0.183 (0.040)	4.98 × 10^−06^	0.068 (0.033)	0.040	2.25 (0.025)	0.156 (0.047)	0.0011	0.038 (0.035)	0.277	2.00 (0.046)
HOMA2-S	−0.080 (0.040)	0.048	0.065 (0.041)	0.111	−2.52 (0.012)	0.157 (0.042)	1.885 × 10^–04^	0.007 (0.038)	0.844	2.65 (0.008)	0.081 (0.068)	0.233	0.151 (0.059)	0.011	−0.78 (0.436)
Triglycerides	0.208 (0.031)	2.44 × 10^−11^	0.120 (0.027)	1.10 × 10^−05^	2.16 (0.031)	0.213 (0.034)	4.29 × 10^−10^	0.204 (0.028)	7.21 × 10^−13^	0.22 (0.827)	0.208 (0.047)	1.06 × 10^−05^	0.249 (0.039)	8.97 × 10^−10^	−0.66 (0.507)
Cholesterol	0.279 (0.033)	5.23 × 10^−16^	0.151 (0.036)	3.58 × 10^−05^	2.58 (0.010)	0.364 (0.032)	4.56 × 10^−27^	0.230 (0.033)	5.00 × 10^−12^	2.93 (0.003)	0.319 (0.045)	1.07 × 10^−11^	0.250 (0.040)	1.35 × 10^−09^	1.13 (0.257)
HDL-cholesterol	0.244 (0.036)	4.28 × 10^−11^	0.242 (0.035)	1.28 × 10^−11^	0.05 (0.956)	0.390 (0.039)	8.14 × 10^−22^	0.251 (0.033)	6.07 × 10^−14^	2.76 (0.006)	0.382 (0.049)	8.90 × 10^−14^	0.237 (0.044)	1.58 × 10^−07^	2.20 (0.028)
LDL-cholesterol	0.319 (0.037)	2.36 × 10^−16^	0.215 (0.050)	1.14 × 10^−07^	1.89 (0.059)	0.425 (0.035)	1.67 × 10^−29^	0.269 (0.037)	2.96 × 10^−12^	3.02 (0.002)	0.324 (0.046)	8.19 × 10^−12^	0.214 (0.039)	8.50 × 10^−08^	1.84 (0.065)
Sons	*N*=363		*N*=342			*N*=342		*N*=314			*N*=253		*N*=205		
Fasting glucose	0.255 (0.040)	5.55 × 10^−10^	0.137 (0.032)	2.250 × 10^–05^	2.32 (0.020)	0.148 (0.037)	7.43 × 10^−05^	0.034 (0.021)	0.117	2.68 (0.007)	0.112 (0.028)	0.0001	0.028 (0.025)	0.208	2.32 (0.020)
Fasting insulin	−0.152 (0.059)	0.01	0.074 (0.060)	0.219	−2.69 (0.007)	0.182 (0.067)	0.007	0.053 (0.062)	0.397	1.41 (0.158)	0.151 (0.089)	0.092	0.099 (0.069)	0.151	0.46 (0.648)
HOMA2-B	−0.047 (0.055)	0.392	0.139 (0.057)	0.014	−2.36 (0.019)	0.203 (0.063)	0.001	0.057 (0.050)	0.256	1.82 (0.068)	0.118 (0.063)	0.063	0.052 (0.053)	0.331	0.80 (0.425)
HOMA2-S	−0.153 (0.059)	0.009	0.066 (0.058)	0.261	−2.64 (0.008)	0.175 (0.067)	0.01	0.054 (0.064)	0.402	1.30 (0.195)	0.139 (0.086)	0.107	0.101 (0.069)	0.146	0.35 (0.729)
Triglycerides	0.241 (0.042)	1.97 × 10^−08^	0.060 (0.038)	0.11	3.22 (0.001)	0.249 (0.042)	6.97 × 10^−09^	0.141 (0.037)	0.0001	1.94 (0.052)	0.214 (0.061)	5.597 × 10^–04^	0.321 (0.057)	9.08 × 10^−08^	−1.27 (0.205)
Cholesterol	0.304 (0.048)	6.13 × 10^−10^	0.099 (0.047)	0.034	3.07 (0.002)	0.382 (0.044)	1.91 × 10^−16^	0.206 (0.045)	7.28 × 10^−06^	2.81 (0.005)	0.311 (0.058)	3.04 × 10^−07^	0.236 (0.054)	2.45 × 10^−05^	0.94 (0.348)
HDL-cholesterol	0.262 (0.049)	1.970 × 10^–07^	0.230 (0.049)	4.350 × 10^–06^	0.46 (0.647)	0.410 (0.056)	3.73 × 10^−12^	0.236 (0.048)	1.34 × 10^−06^	2.35 (0.019)	0.444 (0.066)	2.18 × 10^−10^	0.185 (0.060)	0.002	2.91 (0.004)
LDL-cholesterol	0.297 (0.053)	4.21 × 10^−08^	0.141 (0.054)	0.009	2.06 (0.040)	0.435 (0.050)	3.43 × 10^−16^	0.304 (0.054)	5.89 × 10^−08^	1.77 (0.076)	0.300 (0.056)	2.46 × 10^−07^	0.221 (0.052)	3.07 × 10^−05^	1.04 (0.299)
Daughters	*N*=327		*N*=313			*N*=316		*N*=283			*N*=209		*N*=152		
Fasting glucose	0.319 (0.064)	1.30 × 10^−06^	0.250 (0.056)	1.27 × 10^−05^	0.81 (0.420)	0.359 (0.054)	1.34 × 10^−10^	0.187 (0.032)	2.28 × 10^−08^	2.75 (0.006)	0.044 (0.032)	0.173	0.042 (0.026)	0.113	0.06 (0.954)
Fasting insulin	−0.035 (0.058)	0.549	0.052 (0.059)	0.382	−1.05 (0.296)	0.141 (0.050)	0.004	−0.028 (0.043)	0.517	2.57 (0.010)	0.180 (0.120)	0.137	0.213 (0.111)	0.058	−0.20 (0.840)
HOMA2-B	0.006 (0.060)	0.923	0.114 (0.058)	0.051	−1.29 (0.196)	0.171 (0.050)	0.0007	0.082 (0.043)	0.059	1.36 (0.175)	0.254 (0.070)	4.22 × 10^–04^	0.037 (0.045)	0.412	2.62 (0.009)
HOMA2-S	−0.018 (0.056)	0.751	0.049 (0.058)	0.402	−0.82 (0.410)	0.143 (0.052)	0.006	−0.030 (0.045)	0.5	2.53 (0.011)	0.017 (0.102)	0.864	0.276 (0.101)	0.007	−1.81 (0.071)
Triglycerides	0.159 (0.046)	6.169 × 10^–04^	0.197 (0.039)	8.28 × 10^−07^	−0.62 (0.538)	0.150 (0.055)	0.007	0.275 (0.042)	3.67 × 10^−10^	−1.79 (0.073)	0.246 (0.071)	6.942 × 10^–04^	0.186 (0.050)	2.956 × 10^–04^	0.69 (0.487)
Cholesterol	0.252 (0.047)	2.08 × 10^−07^	0.227 (0.058)	1.097 × 10^–04^	0.33 (0.741)	0.343 (0.047)	4.28 × 10^−12^	0.281 (0.047)	8.26 × 10^−09^	0.93 (0.353)	0.332 (0.073)	1.210 × 10^–05^	0.251 (0.060)	5.160 × 10^–05^	0.86 (0.389)
HDL-cholesterol	0.219 (0.055)	8.47 × 10^−05^	0.261 (0.050)	3.73 × 10^−07^	−0.56 (0.572)	0.381 (0.054)	2.04 × 10^−11^	0.270 (0.045)	6.14 × 10^−09^	1.56 (0.118)	0.261 (0.074)	5.399 × 10^–04^	0.338 (0.066)	1.230 × 10^–06^	−0.77 (0.441)
LDL-cholesterol	0.363 (0.055)	1.86 × 10^−10^	0.316 (0.060)	3.08 × 10^−07^	0.57 (0.567)	0.420 (0.050)	2.56 × 10^−15^	0.233 (0.051)	7.35 × 10^−06^	2.62 (0.009)	0.380 (0.081)	6.62 × 10^−06^	0.176 (0.060)	0.004	2.02 (0.044)

Association between offspring measures with that of each of the parents are presented as β values and SE. Differences between maternal and paternal effects are presented as *Z* values and *p* values. The *Z* value shows the difference between regression coefficients. All regression models were adjusted for age, sex and BMI

MC, mother–child association; FC, father–child association

A contrasting shift from 6 years to 12 years was observed for the paternal and maternal effects in relation to insulin and its indices. For fasting insulin and HOMA2-S, there was a significant negative maternal association at 6 years, which shifted to a significant positive one at 12 years, reflecting a change in the direction of the parent-of-origin effect. For HOMA2-B, there was a stronger positive paternal effect at 6 years, which changed to a stronger positive maternal effect at 12 years and continued to show a maternal effect at 24 years (Figs 2b and 3g, h and Table 2).

In sons, fasting insulin and HOMA2-S showed a significant negative maternal association at 6 years, which shifted to a positive one at 12 years, and the parent-of-origin effects were significant only at 6 years. HOMA2-B showed a paternal bias at 6 years, which shifted to a maternal effect at 12 years. In daughters, a positive maternal bias was seen for fasting insulin and HOMA2-S at 12 years and for HOMA2-B at 24 years (Table 2).

#### Lipid levels

A strong and consistent maternal effect was seen for triglycerides, total cholesterol, HDL-cholesterol and LDL-cholesterol at all timepoints (Figs 2b and i–l). A similar association was seen when analyses were performed separately for sons and daughters (Table 2).

### Longitudinal modelling by mixed-effect models

Given the availability of the measurements across multiple timepoints, we assessed the overall parent-specific effects using longitudinal mixed-effect models. A significant association of anthropometric traits with each of the parents was seen; however, no parent-of-origin effects were observed (ESM Table 6). For cardiometabolic traits, stronger maternal bias was observed for fasting glucose, triglycerides and cholesterol, while stronger paternal bias was observed for fasting insulin, HOMA2-B and HOMA2-S, when all offspring were analysed together, reflecting the pooled effect for each parent for all timepoints combined. Similar associations were also seen for sons separately, with addition of stronger maternal effects for HDL-cholesterol. However, for daughters, stronger maternal bias was seen only for cholesterol (ESM Table 7 and ESM Fig. 2).

### Parent-of-origin effects in the PCS birth cohort

To assess whether similar patterns of parent-of-origin effects could be observed in another birth cohort, we used the data from the cohort in the PCS, comprising approximately 400 trios with follow-up at 4, 8 and 21 years. We used the models described above to assess heritability and parent-of-origin effects on cardiometabolic traits. Anthropometry data were available at 4, 8 and 21 years, and data for other cardiometabolic traits were available at 8 and 21 years.

For all offspring combined, as well as sons and daughters separately, heritability estimates were concordant with those seen in the PMNS for anthropometric and lipid traits. For anthropometric measures, the increasing trend in the paternal effects from 4 to 21 years in the PCS mirrored that in the PMNS from 6 to 24 years. For glucose–insulin traits, heritability estimates were significant for fasting glucose, HOMA2-B and HOMA2-S at 8 years, and that for fasting plasma glucose was significant at 21 years for all offspring combined. For the analysis of sons and daughters separately, heritability estimates were significant for fasting glucose at 8 and 21 years (ESM Table 8).

Some of the parent-of-origin effects in the PMNS were also seen in the PCS. Similar to the PMNS, no parental bias was seen for anthropometric traits, with the exception of a maternal bias for height at 8 years (ESM Table 9). We observed a maternal bias for fasting glucose and fasting insulin at 8 years, similar to that seen in the PMNS at 12 years for all offspring. A similar trend was seen for HOMA2-B and HOMA2-S at 8 years (ESM Table 10).

A consistent maternal bias was seen at 8 and 21 years for cholesterol, and a stronger maternal effect was seen at both timepoints for triglyceride levels for all offspring combined. A stronger maternal effect was also seen for HDL-cholesterol at 21 years for all offspring combined, similar to that seen in the PMNS at 24 years. Similar effects on cholesterol and HDL-cholesterol were also seen for sons and daughters separately (ESM Table 10).

### Genetic parent-of-origin effects in PMNS

#### *KCNQ1* locus

The rs2237892 SNP at the *KCNQ1* locus was previously shown to display parent-of-origin effects on type 2 diabetes risk and insulin secretion [8, 14, 26]. Here we examined whether the same variant showed similar effects on HOMA2-B at 6, 12 and 24 years, and found that the *KCNQ1* variant showed significant parent-of-origin specific associations at 12 and 24 years, with the previously reported type 2 diabetes risk and insulin secretion-lowering maternal allele C lowering (and the alternate T allele increasing) HOMA2-B at 12 and 24 years. Although the same association was not statistically significant at 6 years, the direction of effect was the opposite, similar to that seen in the phenotype correlations (Table 3).

**Table 3:** Genetic parent-of-origin effects of variants at *KCNQ1*, *KLF14* and *MOB2* on relevant cardiometabolic traits

Trait	rsID	Locus	Chr	Position	Ref. allele	Alt.allele	Maternal estimate	Maternal SE	Maternal *p* value	Paternal estimate	Paternal SE	Paternal *p* value	POE estimate	POE SE	POE *p* value
^a^HOMA2-B, 6 years	rs2237892	*KCNQ1*	11	2818521	C	T	−0.200	0.286	0.484	0.240	0.264	0.364	−0.578	0.434	0.183
^a^HOMA2-B, 12 years	rs2237892	*KCNQ1*	11	2818521	C	T	0.238	0.209	0.254	−0.304	0.094	0.001	0.618	0.186	8.597×10^–04^
^a^HOMA2-B, 24 years	rs2237892	*KCNQ1*	11	2818521	C	T	0.333	0.0387	0	0.190	0.157	0.226	0.359	0.020	0
Birthweight	rs2237892	*KCNQ1*	11	2818521	C	T	0.383	0.140	0.006	0.079	0.053	0.134	0.304	0.148	0.040
^b^Birthweight	rs2237892	*KCNQ1*	11	2818521	C	T	0.391	0.143	0.006	0.081	0.050	0.104	0.299	0.171	0.079
^a^Fasting glucose, 6 years	rs2237892	*KCNQ1*	11	2818521	C	T	−2.226	2.675	0.405	−2.59	2.940	0.378	−0.513	5.083	0.920
^a^Fasting glucose, 12 years	rs2237892	*KCNQ1*	11	2818521	C	T	−0.038	0.0275	0.169	0.038	0.045	0.404	−0.079	0.043	0.063
^a^Fasting glucose, 24 years	rs2237892	*KCNQ1*	11	2818521	C	T	−0.081	0.076	0.285	−0.022	0.082	0.784	−0.038	0.131	0.771
^a^Fasting insulin, 6 years	rs2237892	*KCNQ1*	11	2818521	C	T	−0.357	0.352	0.310	0.276	0.297	0.352	−0.875	0.453	0.053
^a^Fasting insulin, 12 years	rs2237892	*KCNQ1*	11	2818521	C	T	0.246	0.359	0.493	−0.343	0.159	0.031	0.694	0.369	0.060
^a^Fasting insulin, 24 years	rs2237892	*KCNQ1*	11	2818521	C	T	0.252	0.209	0.228	0.203	0.137	0.140	0.411	0.358	0.250
^a^Triglycerides, 6 years	rs2237892	*KCNQ1*	11	2818521	C	T	0.0360	0.120	0.764	0.173	0.098	0.077	−0.289	0.062	2.710×10^−06^
^a^Triglycerides, 12 years	rs2237892	*KCNQ1*	11	2818521	C	T	−0.256	0.203	0.209	−0.023	0.149	0.876	−0.288	0.231	0.213
^a^Triglycerides, 24 years	rs2237892	*KCNQ1*	11	2818521	C	T	−0.168	0.179	0.347	0.057	0.169	0.738	−0.353	0.385	0.360
^a^Cholesterol, 6 years	rs2237892	*KCNQ1*	11	2818521	C	T	0.0023	0.105	0.983	0.112	0.095	0.239	−0.227	0.122	0.062
^a^Cholesterol, 12 years	rs2237892	*KCNQ1*	11	2818521	C	T	−0.157	0.088	0.075	0.089	0.099	0.367	−0.235	0.128	0.067
^a^Cholesterol, 24 years	rs2237892	*KCNQ1*	11	2818521	C	T	−0.112	0.086	0.191	−0.013	0.038	0.734	−0.082	0.092	0.372
^a^HDL-C, 6 years	rs2237892	*KCNQ1*	11	2818521	C	T	0.0174	0.143	0.903	0.036	0.139	0.795	−0.213	0.110	0.052
^a^HDL-C, 12 years	rs2237892	*KCNQ1*	11	2818521	C	T	−0.103	0.071	0.149	0.109	0.106	0.307	−0.172	0.067	0.010
^a^HDL-C, 24 years	rs2237892	*KCNQ1*	11	2818521	C	T	0.078	0.078	0.316	−0.020	0.111	0.860	−0.072	0.074	0.333
BMI, birth	rs2237892	*KCNQ1*	11	2818521	C	T	0.918	0.342	0.007	0.367	0.444	0.409	0.486	0.606	0.422
BMI, 6 years	rs2237892	*KCNQ1*	11	2818521	C	T	1.041	0.495	0.035	0.479	0.459	0.297	0.601	0.722	0.406
^a^BMI, 12 years	rs2237892	*KCNQ1*	11	2818521	C	T	0.131	0.056	0.019	0.062	0.110	0.570	0.070	0.116	0.543
^a^BMI, 24 years	rs2237892	*KCNQ1*	11	2818521	C	T	0.163	0.060	0.007	−0.035	0.130	0.785	0.106	0.179	0.555
^a^Triglycerides, 6 years	rs4731702	*KLF14*	7	130748625	C	T	−0.084	0.069	0.224	0.123	0.071	0.083	−0.205	0.078	0.009
^a^Triglycerides, 12 years	rs4731702	*KLF14*	7	130748625	C	T	−0.075	0.070	0.286	0.064	0.061	0.295	−0.141	0.076	0.065
^a^Triglycerides, 24 years	rs4731702	*KLF14*	7	130748625	C	T	−0.132	0.096	0.170	0.104	0.097	0.286	−0.232	0.108	0.032
^a^HDL-C, 6 years	rs4731702	*KLF14*	7	130748625	C	T	0.136	0.050	0.007	0.019	0.050	0.705	0.108	0.058	0.062
^a^HDL-C, 12 years	rs4731702	*KLF14*	7	130748625	C	T	0.100	0.045	0.027	0.020	0.039	0.618	0.084	0.051	0.100
^a^HDL-C, 24 years	rs4731702	*KLF14*	7	130748625	C	T	0.087	0.044	0.048	0.007	0.041	0.857	0.076	0.048	0.112
^a^Cholesterol, 6 years	rs4731702	*KLF14*	7	130748625	C	T	0.027	0.043	0.525	0.020	0.035	0.566	0.007	0.047	0.878
^a^Cholesterol, 12 years	rs4731702	*KLF14*	7	130748625	C	T	0.033	0.033	0.320	−0.004	0.037	0.923	0.035	0.043	0.414
^a^Cholesterol, 24 years	rs4731702	*KLF14*	7	130748625	C	T	0.026	0.036	0.473	0.042	0.041	0.311	−0.018	0.046	0.703
^a^Fat mass, 6 years	rs4731702	*KLF14*	7	130748625	C	T	−0.00	0.057	0.958	0.062	0.061	0.305	−0.055	0.068	0.422
^a^Skinfold, 6 years	rs4731702	*KLF14*	7	130748625	C	T	−0.074	0.031	0.019	0.007	0.032	0.828	−0.075	0.038	0.045
^a^HDL-C, 6 years	rs2334499	*MOB2*	11	1675619	C	T	−0.119	0.056	0.034	−0.029	0.050	0.567	−0.087	0.072	0.223
^a^HDL-C, 12 years	rs2334499	*MOB2*	11	1675619	C	T	−0.104	0.042	0.012	0.027	0.046	0.554	−0.131	0.055	0.018
^a^HDL-C, 24 years	rs2334499	*MOB2*	11	1675619	C	T	−0.110	0.043	0.011	−0.012	0.044	0.789	−0.089	0.053	0.093

All analyses were adjusted for age, sex and BMI

^a^Calculated as log_2_ value

^b^Adjusted for gestational age

rsID, SNP ID; Ref., reference; Alt. alternate; maternal/paternal estimate, β value for the association of maternal allele against reference homozygous genotype for a given trait; maternal/paternal SE, SE value for the association of maternal/paternal allele against reference homozygous genotype for a given trait; maternal/paternal *p* value, *p* value for the association of maternal/paternal allele against reference homozygous genotype for a given trait; POE estimate, parent-of-origin estimate, β value for the association of reciprocal heterozygotes (maternal minor allele vs paternal minor allele) for a given trait; POE SE, SE value for the association of reciprocal heterozygotes (maternal minor allele vs paternal minor allele) for a given trait; POE *p* value, *p* value for the association of reciprocal heterozygotes (maternal minor allele vs paternal minor allele) for a given trait; HDL-C, HDL-cholesterol

We also found significant parental effects on birthweight, triglycerides at 6 years and HDL-cholesterol at 12 years (Table 3).

#### *KLF14* locus

A maternal parent-of-origin effect was seen for the rs4731702 variant at the *KLF14* locus for triglyceride levels at 6 and 24 years. The type 2 diabetes risk-increasing maternal allele C [14] in the *KLF14* variant increased triglyceride levels while the T allele decreased them. The maternal T allele also increased the sum of skinfolds at 6 years (Table 3).

#### *MOB2* locus

The rs2334499 variant at the *MOB2* locus showed significant maternal effects at 6, 12 and 24 years, with significant parent-of-origin effects at 12 years. Previous studies showed that the T allele of the *MOB2* variant increased type 2 diabetes risk when inherited from the father but decreased type 2 diabetes risk when inherited from the mother [14]. In our study, the maternal T allele decreased HDL-cholesterol levels at 6, 12 and 24 years (Table 3).

## Discussion

By harnessing the potential of a birth cohort, we observed strong parent-of-origin effects for birthweight and metabolic traits but only occasionally for postnatal anthropometric traits in a life-course model. These parent-specific effects on clinically relevant phenotypes changed over time for specific cardiometabolic traits, and may have potentially important implications for life-history theory and clinical practice. Analysis of serial measurements cross-sectionally at available timepoints revealed changing parental influences on glucose and insulin metabolism, including insulin secretion and sensitivity, in the offspring. For insulin secretion, there was a transition from a predominantly paternal association in early childhood to a maternal association at pubertal age, whereas, for insulin sensitivity, a significant negative maternal association transitioned to a significantly positive one. Thus, both insulin secretion and action at pubertal age were predominantly associated with maternal phenotype. The maternal effects on lipid traits remained consistent from childhood to adulthood. Longitudinal modelling showed stronger paternal bias for fasting insulin and its indices but maternal bias for glucose and lipids, indicating their cumulative parental influence over time. Previously reported genetic parent-of-origin effects of variants in *KCNQ1* on insulin secretion [26] were replicated in this study at 12 and 24 years, and the direction of effect mirrored that seen in the phenotype associations. The same variants consistently showed maternal effects on lipid levels and on birthweight (which is partly dependent on gestational age at birth). Parent-of-origin effects were also observed for variants at *KLF14* for triglycerides at 6 years and *MOB2* for lipid levels at 6 and 12 years.

Mendelian genetics stipulates an equal contribution from each of the parents to human traits. However, parent-specific influences on metabolic traits, including the beta cell response to oral glucose and insulin action in target tissues, as well as lipid levels, have been previously described in adult offspring from families of patients with type 2 diabetes and cardiovascular diseases [1, 2, 12]. Since these studies were published, the developmental origins of these disorders have been well established, and the strongest window for epigenetic programming is now thought to be periconceptional [35] and before the three germ layers are established (gastrulation) [36]. This suggests that parental influences should be obvious from early life. To this end, we determined the heritability and parent-of-origin effects in a birth cohort that was followed up at regular intervals into young adulthood. As previously reported, these traits were robustly heritable from early childhood as observed when studied in adults [1, 37–44]. Some of these associations were also present in the smaller PCS cohort.

Our results show maternal effects for total and HDL-cholesterol levels, supporting the well-established previous findings [2, 12], and also show that the effects manifest from childhood, albeit with some differences. A stronger correlation for triglyceride levels was reported between mother and daughter in previous studies compared with our study [1, 12]; however, a significant maternal effect was seen in sons at 6 years and in daughters at 12 and 24 years in the PMNS. These maternal effects for lipid traits in the PMNS were also seen in the PCS cohort. Variants in *KLF14* and *MOB2*, which previously were shown to have parent-of-origin effects on type 2 diabetes [14], here showed parent-specific effects on lipid levels, with the risk-increasing allele being associated with lipid levels. These findings provide insights into parental programming of offspring risk of type 2 diabetes.

Several theories have been proposed to explain the evolutionary origins of parent-of-origin effects, which are a consequence of genomic imprinting. Haig and Moore’s kinship theory suggests that imprinting evolved to adjust gene activity, with different allele dosages benefitting maternal and paternal relatives in terms of evolutionary fitness [45, 46]. Day and Bonduriansky’s sexual antagonism and co-adaptation theory suggests that imprinting evolved to shape offspring resemblance to each parent [47, 48]. Wolf and Hager’s maternal–offspring co-adaptation theory proposes that imprinting evolved to favour expression of the fitter allele at a specific gene location [49]. The common feature of these hypotheses is that some processes create a selective asymmetry between the maternally and paternally inherited allele copies at a specific locus, and this causes selection to favour differential expression of the alleles at the same locus [50]. Our results showing a predominantly maternal influence on birthweight and metabolism of major macronutrients (glucose and lipids) during puberty and reproductive age suggests an intergenerational influence on resource allocation towards fecundity, which is a major component of the life-history theory [51].

The transitioning of parental–offspring associations across time from paternal to maternal (supported by genetic associations, e.g. for *KCNQ1* and HOMA2-B) suggests that the effects may be mediated by epigenetics, as the genome remains constant. Epigenetics is a link between the genes and the environment that facilitates the modulation of expression of a particular trait [52, 53]; this kind of programming superimposed on top of the genetic material occurs by means of chemical moieties (e.g. DNA methylation, histone modifications and miRNA), which can alter the way the DNA is read and expressed. Early-life exposures can bring about such epigenetic reprogramming and alter development and function of organs, which, in later life, can increase susceptibility to cardiometabolic disorders [53]. Waterland, Gunasekara and colleagues recently described ‘CoRSIVs’ (correlated regions of systemic interindividual variation) for methylation consistency across tissues representing the three germ layers. These regions were strongly influenced by the periconceptional environment, with some showing epigenetic metastability, and these alterations programmed the risk for future cardiometabolic diseases [54]. *KCNQ1* may well represent an example of such a region [54], as supported by the maternal parent-of-origin effect on birthweight. Furthermore, parent-of-origin effects have been shown to have spatial and temporal effects. Such effects in early life can have implications for development, while in later life they may have implications for function. For example, *KCNQ1* has been shown to have monoallelic expression in fetal islets but expression is biallelic in adults [55]. Functional studies are required to dissect the implications of such dynamic parent-specific effects.

For insulin secretion, the paternal effect in early childhood (6 years) gives way to a maternal effect at 12 years that continues to adulthood (24 years). For fasting insulin and insulin sensitivity, the negative maternal effect at 6 years changed to a positive effect at 12 years. The timing of this transition seemingly spans pubertal age (generally considered to be between 8 and 14 years of age). Metabolism and puberty are strongly interlinked; the link between nutrition and pubertal development requires maintenance of a minimum positive energy balance, especially in girls [56–59], and undernutrition or overnutrition can have a significant impact on the timing and progress of pubertal development and indeed fertility [56, 60]. It may therefore be speculated that parent-of-origin effects accompany pubertal changes given the increased developmental plasticity at this time period [61]. Nevertheless, it remains to be seen whether the shift in parental programming is a cause, consequence or by-product linked to pubertal processes.

It is interesting to note that anthropometric measures do not show a parent-specific association, unlike that seen for metabolic traits. Anthropometric traits are a consequence of genetics and environment, with heritability estimates increasing over time, which may be partially attributed to environmental influences. The reason for the discrepancy between anthropometric and metabolic traits in terms of intergenerational transmission of risk remains uncertain. The life-course theory may explain parents’ changing roles in the allocation of resources for maintenance, growth, reproduction and immunity. Further studies in the purview of evolutionary biology could shed more light on this.

Our study has several limitations. The findings are observational, whereby associations between parental and offspring phenotypes across trajectories of early childhood are examined, and are therefore not causal. Furthermore, this study is based on two birth cohorts from a single city in India, and validation is required in other similar as well as diverse populations to substantiate the findings. Nevertheless, our study is based on birth cohorts with robust study power and extensive follow-up, and therefore has the potential to answer novel questions, as well as providing a context to findings in adult offspring from other populations. The results of genetic and epigenetic studies in family cohorts as well as target tissues will be very useful in unravelling the mechanisms underlying these parental biases and the evolution of parental programming states.

This proof-of-concept study demonstrates parent-of-origin effects on cardiometabolic traits, from birth through childhood and puberty and into adulthood. We extend the life-history theory to demonstrate the life-course evolution of maternal influences on macronutrient metabolism of the offspring. We hypothesise that this indicates serial epigenetic reprogramming (of glucose metabolism) during pubertal and reproductive windows in the life course against the background of persistent maternal genetic effects (on lipid levels). These effects have implications for fecundity and survival of the offspring. Our results have the potential to provide a novel lead into primordial prevention of cardiometabolic diseases.

## Supplementary Information

Below is the link to the electronic supplementary material.

ESM Figures (PDF 221 KB)

ESM Tables (XLSX 51 KB)

## Data Availability

The data that support the findings of this study are not openly available for reasons of sensitivity, and are available from the corresponding author upon reasonable request. Data are stored in controlled-access data storage at the King Edward Memorial Hospital, Pune, India, and Lund University, Malmö, Sweden.

## Abbreviations

GWAS: Genome-wide association study
PCS: Pune Children’s Study
PMNS: Pune Maternal Nutrition Study
