# Supplementary material for: Parent-of-origin effects in the life-course evolution of cardiometabolic traits
Source: Diabetologia. 2025 Apr 2;68(6):1298–314. doi: 10.1007/s00125-025-06396-5 (PMC12069499; doi:10.1007/s00125-025-06396-5)
Supplement: Supplementary file 1 — ESM Figures (PDF 221 KB) [file 125_2025_6396_MOESM1_ESM.pdf]

ESM figure 1: STROBE flow diagram of the Pune Maternal Nutrition Study. Maximum numbers available are mentioned. Not all data may be available on the mentioned numbers.

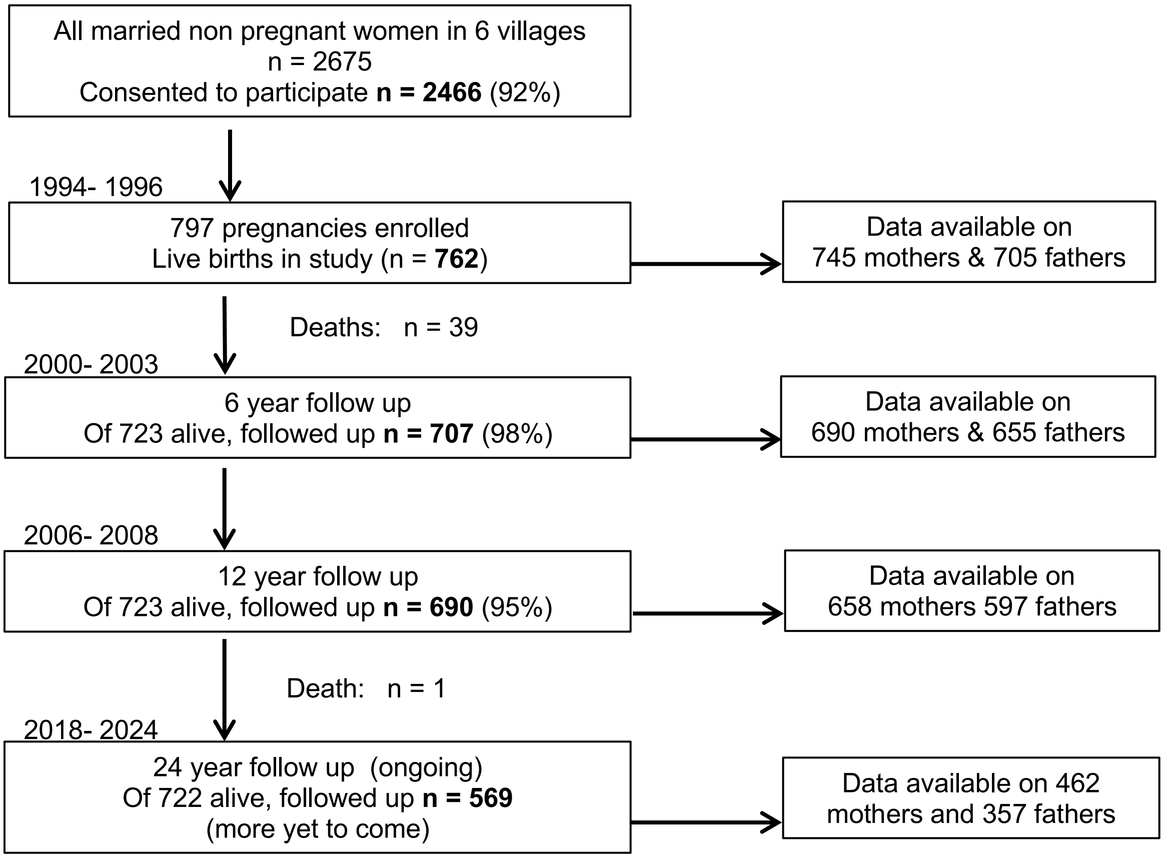

2a Bar graphs of regression coefficients and Z values across time points

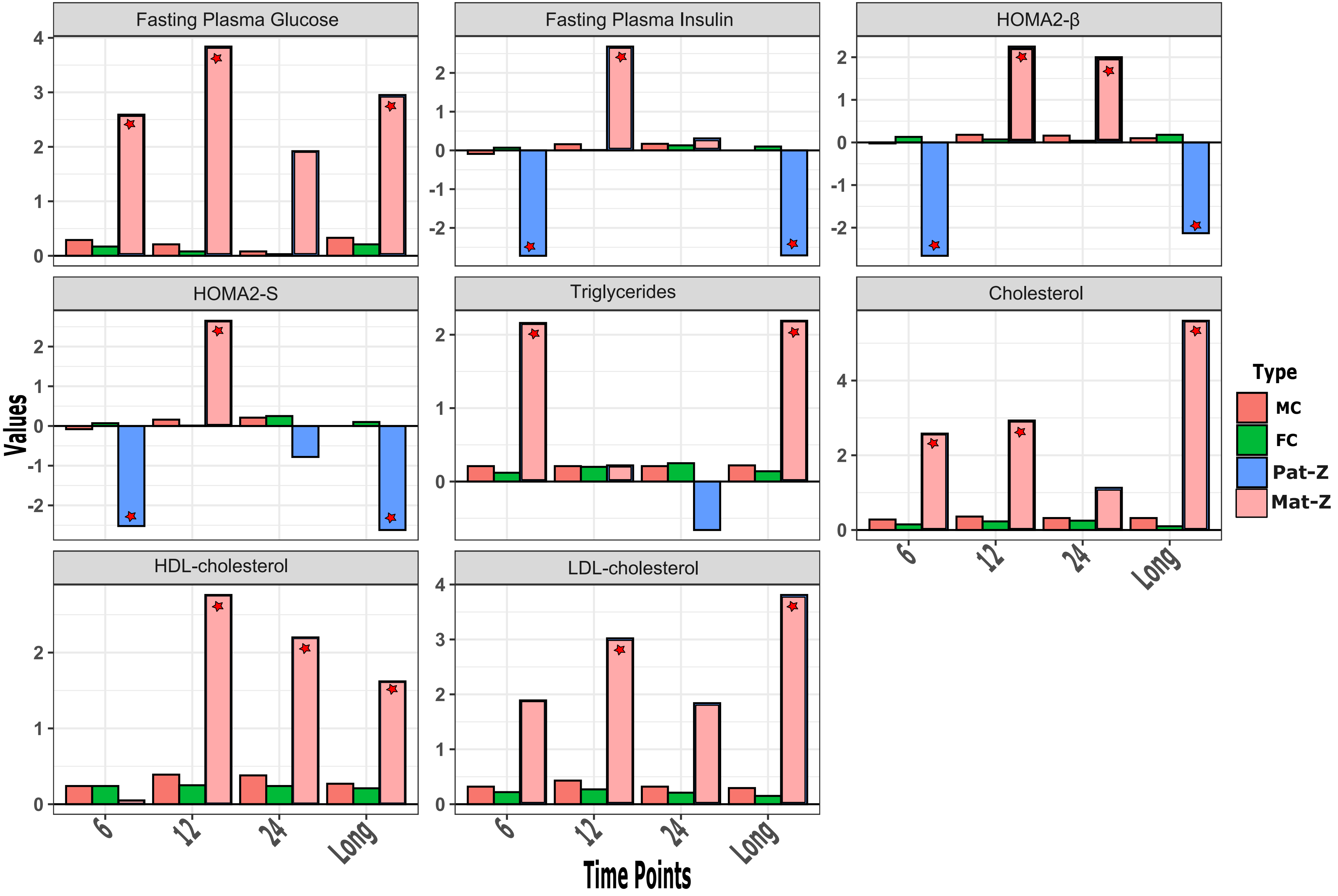

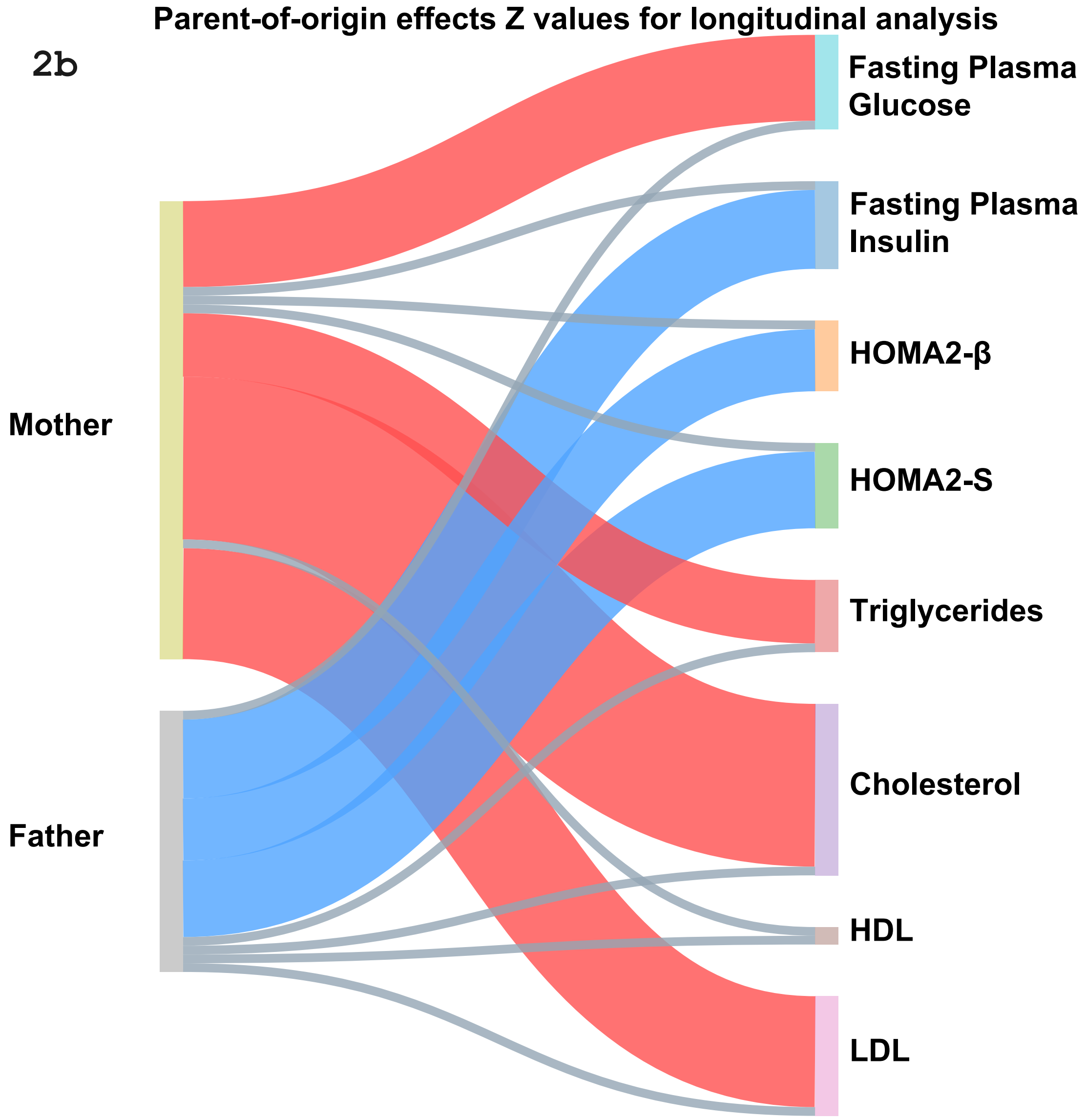
Parent-of-origin effects Z values for longitudinal analysis
2b
Mother
Father
Fasting Plasma Glucose
Fasting Plasma Insulin
HOMA2-β
HOMA2-S
Triglycerides
Cholesterol
HDL
LDL
